# Emerging phenomena in neural networks with dynamic synapses and their computational implications

**DOI:** 10.3389/fncom.2013.00030

**Published:** 2013-04-05

**Authors:** Joaquin J. Torres, Hilbert J. Kappen

**Affiliations:** ^1^Granada Neurophysics Group at Institute “Carlos I” for Theoretical and Computational Physics, University of GranadaGranada, Spain; ^2^Donders Institute for Brain Cognition and Behaviour, Radboud University NijmegenNijmegen, Netherlands

**Keywords:** short-term synaptic plasticity, emergence of dynamic memories, memory storage capacity, criticality in up–down cortical transitions, neural stochastic multiresonances

## Abstract

In this paper we review our research on the effect and computational role of dynamical synapses on feed-forward and recurrent neural networks. Among others, we report on the appearance of a new class of dynamical memories which result from the destabilization of learned memory attractors. This has important consequences for dynamic information processing allowing the system to sequentially access the information stored in the memories under changing stimuli. Although storage capacity of stable memories also decreases, our study demonstrated the positive effect of synaptic facilitation to recover maximum storage capacity and to enlarge the capacity of the system for memory recall in noisy conditions. Possibly, the new dynamical behavior can be associated with the voltage transitions between up and down states observed in cortical areas in the brain. We investigated the conditions for which the permanence times in the up state are power-law distributed, which is a sign for criticality, and concluded that the experimentally observed large variability of permanence times could be explained as the result of noisy dynamic synapses with large recovery times. Finally, we report how short-term synaptic processes can transmit weak signals throughout more than one frequency range in noisy neural networks, displaying a kind of *stochastic multi-resonance.* This effect is due to competition between activity-dependent synaptic fluctuations (due to dynamic synapses) and the existence of neuron firing threshold which adapts to the incoming mean synaptic input.

## 1. Introduction

In the last decades many experimental studies have reported that transmission of information through the synapses is strongly influenced by the recent presynaptic activity in such a way that the postsynaptic response can decrease (that is called *synaptic depression*) or increase (or *synaptic facilitation*) at short time scales under repeated stimulation (Abbott et al., 1997; Tsodyks and Markram, 1997). In cortical synapses it was found that after induction of long-term potentiation (LTP), the temporal synaptic response was not uniformly increased. Instead, the amplitude of the initial postsynaptic potential was potentiated whereas the steady-state synaptic response was unaffected by LTP (Markram and Tsodyks, 1996).

From a biophysical point of view it is well accepted that short-term synaptic plasticity including synaptic depression and facilitation has its origin in the complex dynamics of release, transmission and recycling of neurotransmitter vesicles at the synaptic buttons (Pieribone et al., 1995). In fact, synaptic depression occurs when the arrival of presynaptic action potentials (APs) at high frequency does not allow an efficient recovering at short time scales of the available neurotransmitter vesicles to be released near the cell membrane (Zucker, 1989; Pieribone et al., 1995). This causes a decrease of the postsynaptic response for successive APs. Other possible mechanisms responsible for synaptic depression have been described including feedback activation of presynaptic receptors and from postsynaptic processes such as receptor desensitization (Zucker and Regehr, 2002). On the other hand, synaptic facilitation is a consequence of residual cytosolic calcium—that remains inside the synaptic buttons after the arrival of the firsts APs—which favors the release of more neurotransmitter vesicles for the next arriving AP (Bertram et al., 1996). This increase in neurotransmitters causes a potentiation of the postsynaptic response or synaptic facilitation. It is clear that strong facilitation causes a fast depletion of available vesicles so at the end it also induces a strong depressing effect. Other possible mechanisms responsible for short-term synaptic plasticity include, for instance, glial-neuronal interactions (Zucker and Regehr, 2002).

In the two seminal papers (Tsodyks and Markram, 1997) and (Abbott et al., 1997) a simple phenomenological model has been proposed based in these biophysical principles which nicely fits the evoked postsynaptic responses observed in cortical neurons. The model is characterized by three variables *x*_*j*_(*t*), *y*_*j*_(*t*), *z*_*j*_(*t*) that follow the dynamics

(1) $$\begin{array}{l} \frac{dx_{j}(t)}{dt}=\frac{z_{j}(t)}{\tau_{\text{rec}}}-U_{j}\cdot x_{j}(t)\cdot \delta (t-t_{\text{sp}}^{j})\\ \frac{dy_{j}(t)}{dt}=\frac{-y_{j}(t)}{\tau_{\text{in}}}+U_{j}\cdot x_{j}(t)\cdot \delta (t-t_{\text{sp}}^{j})\\ \frac{dz_{j}(t)}{dt}=\frac{y_{j}(t)}{\tau_{\text{in}}}-\frac{z_{j}(t)}{\tau_{\text{rec}}} \end{array}$$

where *y*_*j*_(*t*) is the fraction of neurotransmitters which is released into the synaptic cleft after the arrival of an AP at time *t*^*j*^_sp_, *x*_*j*_(*t*) is the fraction of neurotransmitters which is recovered after previous arrival of an AP near the cell membrane and *z*_*j*_(*t*) is the fraction of inactive neurotransmitters. The model assumes conservation of the total number of neurotransmitter resources in time so one has *x*_*j*_(*t*) + *y*_*j*_(*t*) + *z*_*j*_(*t*) = 1. The released neurotransmitter inactivates with time constant τ_in_ and the inactive neurotransmitter recovers with time constant τ_rec_. The synaptic current received by a postsynaptic neuron from its neighbors is then defined as *I*_*i*_(*t*) = ∑_*j*_*A*_*ij*_*y*_*j*_(*t*) where *A*_*ij*_ represents the maximum synaptic current evoked in the postsynaptic neuron *i* by an AP from presynaptic neuron *j* which in cortical neurons is around 40 *pA* (Tsodyks et al., 1998).

For constant release probability *U*_*j*_, the model describes the basic mechanism of synaptic depression. The model is completed to account for synaptic facilitation by considering that *U*_*j*_ increases in time to its maximum value *U* as the consequence of the residual cytosolic calcium that remains after the arrival of very consecutive APs, and follows the dynamics

(2) $$\frac{dU_{j}(t)}{dt}=\frac{\left[U-U_{j}(t)\right]}{\tau_{\text{fac}}}+U\cdot [1-U_{j}(t)]\cdot \delta (t-t_{\text{sp}}^{j}).$$

Short term synaptic plasticity has profound consequences on information transmission by individual neurons as well as on network functioning and behavior. Previous works have shown this fact on both feed-forward and recurrent networks. For instance, in feed-forward networks activity-dependent synapses act as non-linear filters in supervised learning paradigms (Natschläger et al., 2001), being able to extract statistically significant features from noisy and variable temporal patterns (Liaw and Berger, 1996).

For recurrent networks, several studies revealed that populations of excitatory neurons with depressing synapses exhibit complex regimes of activity (Senn et al., 1996; Tsodyks et al., 1998, 2000; Bressloff, 1999; Kistler and van Hemmen, 1999), such as short intervals of highly synchronous activity (population bursts) intermittent with long periods of asynchronous activity, as is observed in neurons throughout the cortex (Tsodyks et al., 2000). Related with this, it was proposed (Senn et al., 1996, 1998) that synaptic depression may serve as a mechanism for rhythmic activity and central pattern generation. Also, recent studies on rate models have reported the importance of dynamic synapses in the emergence of persistent activity after removal of stimulus which is the base of the so called working memories (Barak and Tsodyks, 2007), and in particular it has been also reported the relevant role of synaptic facilitation, mediated by residual calcium, as the main responsible for appearance of working memories (Mongillo et al., 2008).

All these phenomena have stimulated much research to elucidate the effect and possible functional role of short term synaptic plasticity. In this paper we review our own efforts over the last decade in this research field. In particular, we have demonstrated both theoretically and numerically the appearance of different non-equilibrium phases in attractor networks as the consequence of the underlying noisy activity in the network and of the existence of synaptic plasticity (see section 2). The emergent phenomenology in such networks includes a high sensitivity of the network to changing stimuli and a new phase in which dynamical attractors or dynamical memories appear with the possibility of regular and chaotic behavior and rapid “switching” between different memories (Pantic et al., 2002; Cortes et al., 2004, 2006; Torres et al., 2005, 2008; Marro et al., 2007). The origin of such new phases and the extraordinary sensibility of the system to varying inputs—even in the memory phase—is precisely the “fatigue” of synapses due to heavy presynaptic activity competing with different sources of noise which induces a destabilization of the regular stable memory attractors. One of the main consequences of this behavior is the strong influence of short-term synaptic plasticity on storage capacity of such networks (Torres et al., 2002; Mejias and Torres, 2009) as we will explain in section 3.

The switching behavior is characterized by a characteristic time scale during which the memory is retained. The distribution of time scale depends in a complex way on the parameters of the dynamical synapse model and is the result of a phase transition. We have investigated the conditions for the appearance of power-law behavior in the probability distribution of the permanence times in the Up state, which is a sign for criticality (see section 4). This dynamical behavior has been associated (Holcman and Tsodyks, 2006) to the empirically observed transitions between states of high activity (Up states) and low activity (Down states) in the mammalian cortex (Steriade et al., 1993a,b).

The enhanced sensibility of neural networks with dynamic synapses to external stimuli could provide a mechanism to detect relevant information in weak noisy external signals. This can be viewed as a form of *stochastic resonance* (SR), which is the general phenomenon that enhances the detection by a non-linear dynamical system of weak signals in the presence of noise. Recent experiments in auditory cortex have shown that synaptic depression improves the detection of weak signals through SR for a larger noise range (Yasuda et al., 2008). In a feed-forward network model of spiking neurons, we have modeled these experimental findings (Mejias and Torres, 2011; Torres et al., 2011). We demonstrated theoretically and numerically that, in fact, short-term synaptic plasticity together with non-linear neuron excitability induce a new type of SR where there are multiple noise levels at which weak signals can be detected by the neuron. We denoted this novel phenomenon by *bimodal stochastic resonances* or *stochastic multiresonances* (see section 5) and, very recently, we have proved that this intriguing phenomenon not only occurs in feed-forward neural networks but also in recurrent attractor networks (Pinamonti et al., 2012).

## 2. Appearance of dynamical memories

In this section we review our work on the appearance of dynamical memories in attractor neural networks with dynamical synapses as originally reported in (Pantic et al., 2002; Torres et al., 2002, 2008; Mejias and Torres, 2009). For simplicity and in order to obtain straightforward mean-field derivations we have considered the case of a network of *N* binary neurons (Hopfield, 1982; Amit, 1989). However, we emphasize that the same qualitative behavior emerges in networks of integrate and fire (IF) neurons (Pantic et al., 2002).

Each neuron in the network, whose state is *s*_*i*_ = 1, 0 depending if the neuron is firing or not an AP, receives at time *t* from its neighbor neurons a total synaptic current, or local field, given by

(3) $$h_{i}(t)=\sum_{j}\omega_{ij}(t)s_{j}(t)$$

where ω_*ij*_(*t*) is the synaptic current received by the postsynaptic neuron *i* from the presynaptic neuron *j* when this fires an AP (*s*_*j*_(*t*) = 1). If the synaptic current to neuron *i*, *h*_*i*_(*t*), is larger than some neuron threshold value θ_*i*_, neuron *i* fires an AP with a probability that depends on the intrinsic noise present in the network. The noise is commonly modeled as a thermal bath at temperature *T*. We assume parallel dynamics (Little dynamics) using the probabilistic rule

(4) $$\text{Prob}(s_{i}(t+1)=\sigma )=\frac{1}{2}+\left(\sigma -\frac{1}{2}\right)\tan h[2T^{-1}(h_{i}(t)-\theta_{i})]$$

with σ = 1,0.

To account for short-term synaptic plasticity in the network we consider

(5) $$\omega_{ij}(t)={\bar{\omega }}_{ij}D_{j}(t)F_{j}(t)$$

where *D*_*j*_(*t*) and *F*_*j*_(*t*) are dynamical variables representing synaptic depression and synaptic facilitation mechanisms. The constants ${\bar{\omega }}_{ij}$ denote static maximal synaptic conductances, that contain information concerning a number *P* of random patterns of neural activity, or *memories*, ξ^μ^ ≡ {ξ^μ^_*i*_ = 1,0; *i* = 1,…, *N*, μ = 1, …, *P*} previously learned and stored in the network. Such static memories can be achieved in actual neural systems by LTP or depression of the synapses due to network stimulation with these memories. For concreteness, we assume here that these weights are the result of a Hebbian-like learning process that takes place on a time scale that is long compared to the dynamical time scales of the neurons and the dynamical synapses. The Hebbian learning takes the form

(6) $${\bar{\omega }}_{ij}=\frac{1}{Na(1-a)}\sum_{\mu =1}^{P}\left(\xi_{i}^{\mu }-a)(\xi_{j}^{\mu }-a\right)\;{\bar{\omega }}_{ii}=0,$$

also known as the *covariance learning rule*, with *a* = 〈ξ^μ^_*i*_〉 representing the mean level of activity in the patterns. **I**t is well-known that a recurrent neural network with synapses (Equation 6) acts as an *associative memory* (Amit, 1989). That is, the stored patterns ξ^μ^ become local minima of the free-energy and within the basin of attraction of each memory, the neural dynamics (Equation 4) drives the network activity toward this memory. Thus, appropriate stimulation of (a subset of) neurons that are active in the stored pattern initiates a memory recall process in which the network converges to the memory state.

To model the dynamics of the synaptic depression *D*_*j*_(*t*) and facilitation *F*_*j*_(*t*), we simplify the phenomenological model of dynamic synapses described by Equations (1, 2), taking into account that in actual neural systems such as the cortex τ_in_ « τ_rec_, which implies that *y*_*i*_(*t*) = 0 for most of the time and only at the exact point at which the AP arrives has a non-zero value *y*_*j*_(*t*_sp_) = *x*_*j*_(*t*_sp_) *U*_*j*_(*t*_sp_). Thus, the synaptic current evoked in the postsynaptic neuron *i* by a presynaptic neuron *j* every time it fires is approximatively *I*_*ij*_(*t*) = *A*_*ij*_ *x*_*j*_(*t*^*j*^_sp_) *U*_*j*_(*t*^*j*^_sp_) which has the form given by Equation (5) with ${\bar{\omega }}_{ij}=A_{ij}$, *D*_*j*_(*t*) ≡ *x*_*j*_(*t*) and *F*_*j*_(*t*) ≡ *U*_*j*_(*t*). We set *U* = 1 without loss of generality in order to have *D*_*j*_(*t*) = *F*_*j*_(*t*) = 1 ∀*j*, *t* for τ_rec_, τ_fac_ « 1, that corresponds to the well know limit of *static synapses* without depressing and facilitating mechanism. In this limit, in fact, one recover the classical Amari–Hopfield model of associative memory (Amari, 1972; Hopfield, 1982) when one chooses the neuron thresholds as

(7) $$\theta_{i}=\frac{1}{2}\sum_{j}{\bar{\omega }}_{ij}.$$

It is important to point out that due to the discrete nature of the probabilistic neuron dynamics (Equation 4) together with the approach τ_in_ « τ_rec_, only discrete versions of the dynamics for *x*_*i*_(*t*) and *U*_*i*_(*t*) [see for instance (Tsodyks et al., 1998)] are needed here, namely

(8) $$\begin{array}{l} x_{j}(t+1)=x_{j}(t)+\frac{1-x_{j}(t)}{\tau_{\text{rec}}}-U_{j}(t)\cdot x_{j}(t)\cdot s_{j}(t)\\ U_{j}(t+1)=U_{j}(t)+\frac{\left[U-U_{j}(t)\right]}{\tau_{\text{fac}}}+U\cdot [1-U_{j}(t)]\cdot s_{j}(t).\;\;\;\;\; \end{array}$$

Equations (4–8) completely define the dynamics of the network. Note, that in the limit of τ_rec_, fac → 0 the model reduces to the standard Amari–Hopfield model with static synapses.

To numerically and analytically study the emergent behavior of this attractor neural network with dynamical synapses, it is useful to measure the degree of correlation between the current network state **s** ≡ {*s*_*i*_; *i* = 1, …, *N*} and each one of the stored patterns ξ^μ^ by mean of the overlap function

(9) $$m^{\mu }(s)=\frac{1}{Na(1-a)}\sum_{i}\left(\xi_{i}^{\mu }-a\right)s_{i}.$$

**Figure 1:**
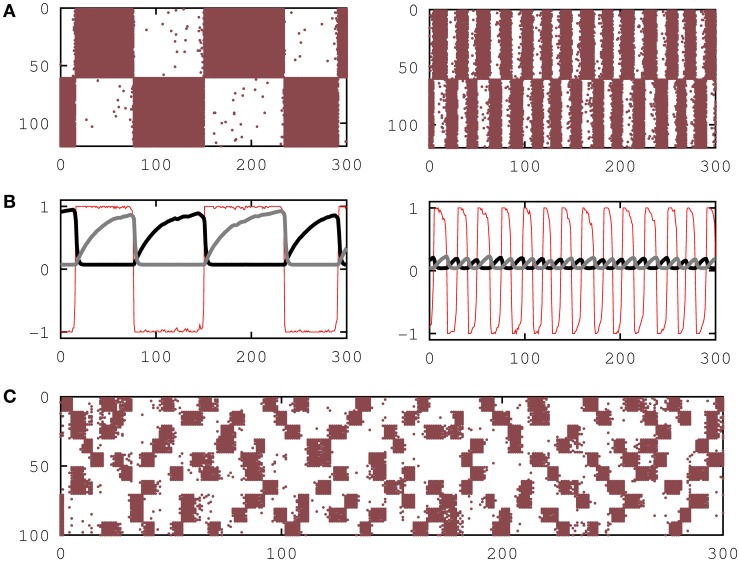
**Emergence of dynamic memories in attractor neural networks. (A)** Raster plots showing the switching behavior of the network neural activity between one activity pattern and its anti-pattern for τ_rec_ = 26 and *T* = 0.025 (left) and for τ_rec_ = 50 and *T* = 0.025 (right), respectively. **(B)** The behavior of the overlap function *m*^μ^(**s**) (thin red line), the mean recovering variable *x*^μ^_+_ of active neurons in the pattern (thick black line) and the mean recovering variable *x*^μ^_−_ of not active neurons in the pattern (thick gray line) are plotted for the two cases depicted in **(A)**. **(C)** Raster plot that shows the emergence of dynamic memories when 10 activity patterns are stored in the synapses for τ_rec_ = 50. In all panels the firing threshold was set to θ_*i*_ = 0, and the network size was *N* = 120 in **(A)** and **(B)** and *N* = 100 in **(C)**.

Monte Carlo simulations of the network storing a small number of random patterns (loading parameter α ≡ *P*/*N* → 0), each pattern having 50% active neurons (*a* = 0.5), no facilitation (*U*_*j*_(*t*) = 1) and an intermediate value of τ_rec_ is shown in Figures 1A,B. It shows a new phase where dynamical memories characterized by quasi-periodic switching of the network activity between pattern (ξ^μ^) and anti-pattern (**1** − ξ^μ^) configurations appear. For lower values of τ_rec_ the network reduces to the attractor network with static synapses and shows the emergence of the traditional *ferromagnetic* or associative memory phase at relatively low *T*, where network activity reaches a steady state that is highly correlated with one of the stored patterns, and a *paramagnetic* or no-memory phase at high *T* where the network activity reaches a highly fluctuating disordered steady state.

**Figure 2:**
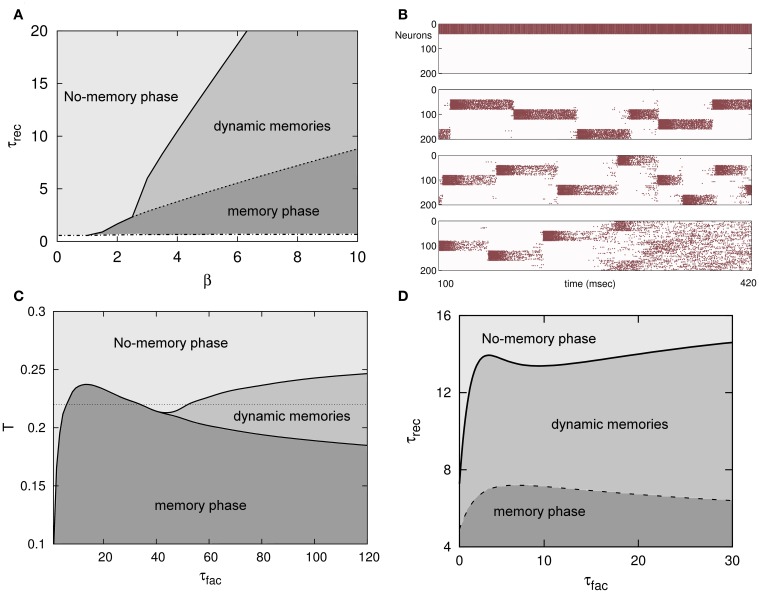
**(A)** Phase diagram (τ_rec_, β ≡ *T*^−1^) of an attractor binary neural network with depressing synapses for α = 0. A new phase in which dynamical memories appear—with the network activity switching between the different memory attractors—emerges between the traditional memory and no-memory phases that characterize the behavior of attractors neural networks with static synapses. **(B)** The emergent behavior depicted in **(A)** is robust when a more realistic attractor network of IF neurons and more stored patterns are considered (5 in this simulation). From top to bottom, the behavior of the network activity for τ_rec_ = 0, 300, 800 and 3000 ms is depicted, respectively. For some level of noise the network activity pass from the memory phase to the dynamical phase and from this to the no-memory phase when τ_rec_ is increased. **(C)** Phase diagram (*T*, τ_fac_) for τ_rec_ = 3 and *U* = 0.1 of an attractor binary neural network with short-term depression and facilitation mechanisms in the synapses and α = 0. **(D)** Phase diagram (τ_rec_, τ_fac_) for *T* = 0.1 and *U* = 0.1 in the same system than in **(C)**. In both, **(C,D)**, the diagrams depict the appearance of the same memory, oscillatory and no-memory phases than in the case of depressing synapses. The transition lines between different phases, however, show here a clear non-linear and non-monotonic dependence with relevant parameters consequence of the non-trivial competition between depression and facilitation mechanisms. This is very remarkable in **(C)** where for a given level of noise, namely *T* = 0.22 (horizontal dotted line), the increase of facilitation time constant τ_fac_ induces the transition of the activity of the network from a no-memory state to a memory state, from this one to a no-memory state again, and finally from this last to an oscillatory regime.

The Figure 1C shows simulation results of a network with *P* = 10 patterns and *a* = 0.1, demonstrating that switching behavior is also obtained for relatively large number of patterns and sparse network activity. Figure 2B shows that the switching behavior is not an artifact of the binary neuron dynamics and is also obtained in a network of more realistic networks of spiking integrate-and-fire neurons.

All time constants, such τ_rec_ or τ_fac_ are given in units of Monte Carlo steps (MCS) a temporal unit that in actual systems can be associated, for instance, with the duration of the refractory period and therefore of order of 5 ms.

In the limit of *N* → ∞ (thermodynamic limit) and α → 0 (finite number of patterns) the emergent behavior of the model can be analytically studied within a standard mean field approach [see for details (Pantic et al., 2002; Torres et al., 2008)]. The dynamics of the system then is described by a 6*P*-dimensional discrete map

(10) $$v_{t+1}=ℱ(v_{t})$$

where ℱ is a 6*P*-dimensional non-linear function of the order parameters

(11) $$\begin{array}{l} v_{t}\equiv \{m_{+}^{\mu }(t),m_{-}^{\mu }(t),x_{+}^{\mu }(t),x_{-}^{\mu }(t),U_{+}^{\mu }(t),U_{-}^{\mu }(t);\\ \;\mu =1,\ldots ,P\} \end{array}$$

that are averages of the microscopic dynamical variables over the sites that are active and quiescent, respectively, in a given pattern μ, that is

(12) $$\begin{array}{l} c_{+}^{\mu }(t)\equiv \frac{1}{Na}\sum_{i\;\in Act(\mu )}c_{i}(t),\\ c_{-}^{\mu }(t)\equiv \frac{1}{N(1-a)}\sum_{i\notin Act(\mu )}c_{i}(t), \end{array}$$

with *c*_*i*_(*t*) being *m*_*i*_(*t*), *x*_*i*_(*t*), and *U*_*i*_(*t*), respectively.

Local stability analysis of the fixed point solutions of the dynamics (Equation 10) shows that, similarly to the Amari–Hopfield standard model and in agreement with Monte Carlo simulations described above, the stored memories ξ^μ^ are stable attractors in some regions of the space of relevant parameters, such as *T*, *U*, τ_rec_, and τ_fac_. Varying these parameters, there are, however, some critical values for which the memories destabilize and an oscillatory regime, in which the network visits different memories, can emerge. These critical values are depicted in Figures 2A,C,D in the form of transition lines between phases or dynamical behaviors in the system. For instance, for only depressing synapses (τ_fac_ = 0, *U*_*j*_(*t*) = 1), there is a critical monotonic line τ^*^_rec_(*T*^−1^), as in a second order phase transition, separating the no-memory phase and the oscillatory phase (solid line in Figure 2A) where oscillations start to appears with small amplitude as in a supercritical Hopf bifurcation. Also there is a transition line τ^**^_rec_(*T*^−1^), also monotonic, between the oscillatory phase and the memory phase which occurs sharply as in a first order phase transition (dashed line in Figure 2A). When facilitation is included, the picture is more complex, although similar critical and sharp transitions lines appear separating the same phases. Now, however, the lines separating different phases are non-monotonic and highly non-linear which shows the competition between *a priori* opposite mechanisms, depressing and facilitating, as is depicted in Figures 2C,D. In fact, among other features, synaptic depression induces fatigue at the synapses which destabilizes the attractors, and synaptic facilitation allows a fast access to the memory attractors and to stay there during a shorter period of time (Torres et al., 2008). As in Figure 1, in all phase diagrams appearing in Figure 2, τ_rec_ and τ_fac_ are given in MCS units (see above) with a value for that temporal unit of around the typical duration of the refractory period in actual neurons (~5 ms).

The attractor behavior of the recurrent neural network has the important property to complete a memory based on partial or noisy stimulus information. In this section we have seen that memories that are stable with static synapses become meta-stable with dynamical synapses, inducing a switching behavior among memory patterns in the presence of noise. In this manner, dynamic synapses provide the associative memory with a natural mechanism to dissociate from a memory in order to associate with a new memory pattern. In contrast, with static synapses the network would stay in the stable memory state forever, preventing recall of new memories. Thus, dynamic synapses change stable memories into meta-stable memories for certain ranges of the parameters.

## 3. Storage capacity

It is important to analyze how short-term synaptic plasticity affects the maximum number of patterns of neural activity the system is able to store and efficiently recall, that is, the so called *maximum storage capacity*. In a recent paper we have addressed this important issue using a standard mean field approach in the model described by Equations (3–8) when it stored *P* = α *N* activity random patterns with α > 0 and *N* → ∞, *a* = 1/2 and in the absence of noise (*T* = 0). In fact, for very low temperature (*T* « 1), redefining the overlaps as $M^{\nu }\equiv m^{\nu }-\frac{1}{N}\sum_{i}\left(2\xi_{i}^{\nu }-1\right)\equiv m^{\nu }-B^{\nu }$ and assuming steady-state conditions in which there is only one pattern (condensed pattern) with overlap $M\equiv M^{1}\sim O(1)$ and the remaining patters $M^{\nu }\sim O(1/\sqrt{N}),\nu =2,\ldots ,P$, it is straightforward (Hertz et al., 1991) to see that the steady state of the system is described by the set of mean field equations

(13) $$\begin{array}{l} M=\frac{1}{N}\sum_{i}\tan h\left[\beta \left(\frac{\gamma^{\prime }}{1+\gamma \gamma^{\prime }}M+\zeta_{i}\right)\right]\\ \;q=\frac{1}{N}\sum_{i}\tan h^{2}\left[\beta \left(\frac{\gamma^{\prime }}{1+\gamma \gamma^{\prime }}M+\zeta_{i}\right)\right]\\ \;r=\frac{q}{{\left(1-\beta \frac{\gamma^{\prime }}{1+\gamma \gamma^{\prime }}(1-q)\right)}^{2}} \end{array}$$

where γ ≡ *U*τ_rec_, $\gamma^{\prime }\equiv \frac{1+\tau_{\text{fac}}}{1+U\tau_{\text{fac}}}$, $q\equiv \frac{1}{N}\sum_{i}\tan h^{2}[2\beta (h_{i}(t)-\theta_{i})]$ is the spin-glass order parameter, $r=\frac{1}{\alpha }\sum_{\nu \neq 1}{\left(M^{\nu }\right)}^{2}$ is the pattern interference parameter and

$$\zeta_{i}\equiv \sum_{\nu \neq 1}\left(2\xi_{i}^{1}-1)(2\xi_{i}^{\nu }-1\right)\left[\frac{\gamma^{\prime }}{1+\gamma \gamma^{\prime }}M^{\nu }+\left(1-\frac{\gamma^{\prime }}{1+\gamma \gamma^{\prime }}\right)B^{\nu }\right]$$

which in the limit of *N* → ∞ becomes a Gaussian variable

$$\zeta \approx \frac{\gamma^{\prime }}{1+\gamma \gamma^{\prime }}{\left(\alpha r+\alpha {\left(\frac{1+\gamma \gamma^{\prime }-\gamma^{\prime }}{\gamma^{\prime }}\right)}^{2}\right)}^{1/2}z$$

where *z* is a random normal-distributed variable *N*[0,1]—see details in (Mejias and Torres, 2009). Then, the $\frac{1}{N}\sum_{i}$ appearing in Equation (13) becomes an average over 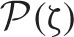. Using standard techniques in the limit *T* = 0 (Hertz et al., 1991), the set of the resulting three mean-field equations reduces to a single mean-field equation which gives the maximum number of patterns that the system is able to store and retrieve, namely (see mathematical details in Mejias and Torres, 2009)

(14) $$y\left[\sqrt{2\alpha {\left(\frac{1+\gamma \gamma^{\prime }-\gamma^{\prime }}{\gamma^{\prime }}\right)}^{2}}+\frac{2}{\sqrt{\pi }}\exp(-y^{2})\right]=\text{erf}(y)$$

where $y\equiv M/\sqrt{\left(2\alpha r+2\alpha {\left(\frac{1+\gamma \gamma^{\prime }-\gamma^{\prime }}{\gamma^{\prime }}\right)}^{2}\right)}$ with *M* being the overlap of the current state of the network activity with the pattern that is being retrieved. The Equation (14) has a trivial solution *y* = 0 (*M* = 0). Non-zero solutions (with non-zero overlap *M*) exist for α less than some critical α, which defines the maximum storage capacity of the system α_*c*_.

**Figure 3:**
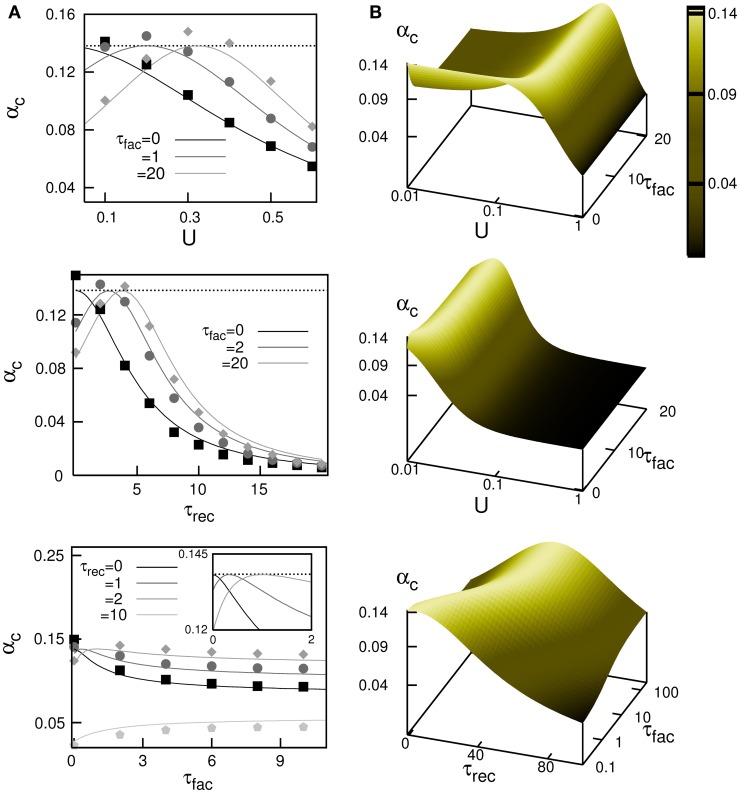
**Maximum storage capacity obtained in attractor neural networks with dynamic synapses with both depressing and facilitating mechanisms. (A)** Behaviour of α_*c*_ as a function of *U*, τ_rec_ and τ_fac_. The solid lines correspond the theoretical prediction from the mean field Equation (14) and symbols are obtained from Monte Carlo simulations. From top to bottom, it is shown α_*c*_(*U*) for τ_rec_ = 2 and different values of τ_fac_, α_*c*_(τ_rec_) for *U* = 0.2 and different values of τ_fac_ and α_*c*_(τ_fac_) for *U* = 0.2 and different values of τ_rec_, respectively. The horizontal dotted lines correspond to the static synapses limit α_*c*_ ≈ 0.138. **(B)** Mean-field results from Equation (14) for the dependence of α_*c*_ for different combinations of relevant parameters. This corresponds—from top to bottom—to the surfaces α_*c*_(*U*, τ_fac_) for τ_rec_ = 2, α_*c*_(*U*, τ_fac_) for τ_rec_ = 50 and α_*c*_(τ_rec_, τ_fac_) for *U* = 0.02. In all panels, τ_rec_ and τ_fac_ are given in MCS units that can be associated to a value of 5 ms if one assumes that a MCS corresponds to the duration of the refractory period in actual neurons.

A complete study of the system by means of Monte Carlo simulations (in a network with *N* = 3000 neurons) has demonstrated the validity of this mean field result and is depicted in Figure 3A. The figure shows the behavior of α_*c*_ obtained from Equation (14) (different solid lines), when some relevant parameters of the synapse dynamics are varied, and it is compared with the maximum storage capacity obtained from the Monte Carlo simulations (different symbols). The most remarkable feature is that in the absence of facilitation the storage capacity decreases when the level of depression increases (that is, large release probability *U*, or large recovering time τ_rec_); see black curves in the top and middle panels of Figure 3A. This decrease is caused by the loss of stability of the memory fixed points of the network due to depression. Facilitation (see dark and light gray curves) allows to recover the maximal storage capacity of static synapses, which is the well know limit α_*c*_ ≈ 0.14 (dotted horizontal line), in the presence of some degree of synaptic depression. In general the competition between synaptic depression and facilitation induces a complex non-linear and non-monotonic behavior of α_*c*_ for different synaptic dynamics parameters as is shown in different panels of Figure 3B. In general, large values of α_*c*_ appear for moderate values of *U* and τ_rec_, and large values of τ_fac_. These values qualitatively agree with those described in facilitating synapses in some cortical areas, where *U* is lower than in the case of depressing synapses and τ_rec_ is several times lower than τ_fac_ (Markram et al., 1998). Note that facilitation or depression never increases the storage capacity of the network above the maximum value α_*c*_ ≈ 0.14.

## 4. Criticality in up–down transitions

In a recent paper (Holcman and Tsodyks, 2006), the emergent dynamic memories described in section 2 that result from short-term plasticity have been related to the voltage transitions observed in cortex between a high-activity state (the Up state) and a low-activity state (the Down state). These transitions have been observed in simultaneous individual single neuron recordings as well as in local field measurements.

Using a simple but biologically plausible neuron and synapse model similar to the models described in sections 1 and 2, we have theoretically studied the conditions for the emergence of this intriguing behavior, as well as their temporal features (Mejias et al., 2010). The model consists of a simple stochastic bistable rate model which mimics the average dynamics of a population of interconnected excitatory neurons. The neural activity is summarized by a single activity ν(*t*), whose dynamics follows a stochastic mean field equation

(15) $$\tau_{\nu }\frac{d\nu (t)}{dt}=-\nu (t)+\nu_{m}S[J\nu (t)x(t)-\theta ]+\zeta (t)$$

where τ_ν_ is the time constant for the neuron dynamics, ν_*m*_ is the maximum synaptic input to the neuron population, *J* is the (static) synaptic strength and θ is the neuron threshold. The function S[X] is a sigmoidal function which models the excitability of neurons in the population.

The synaptic input from other neurons is modulated by a short-term dynamic synaptic process *x*(*t*) which satisfies the stochastic mean field equation

(16) $$\frac{dx(t)}{dt}=\frac{1-x(t)}{\tau_{r}}-Ux(t)\nu (t)+\frac{D}{\tau_{r}}\xi (t).$$

The parameters τ_*r*_, *U* and *D* are, respectively, the recovery time constant for the stochastic short-term synaptic plasticity mechanism, a parameter related with the reliability of the synaptic transmission (the average release probability in the population) and the amplitude of this synaptic noise. The explanation of each term appearing in the rhs of Equation (16) is the following: the first term accounts for the slow recovery of neurotransmitter resources, the second term represents a decrease of the available neurotransmitter due to the level of activity in the population and the third term is a noise term that accounts for all possible sources of noise affecting transmission of information at the synapses of the population and that remains at the mesoscopic level.

**Figure 4:**
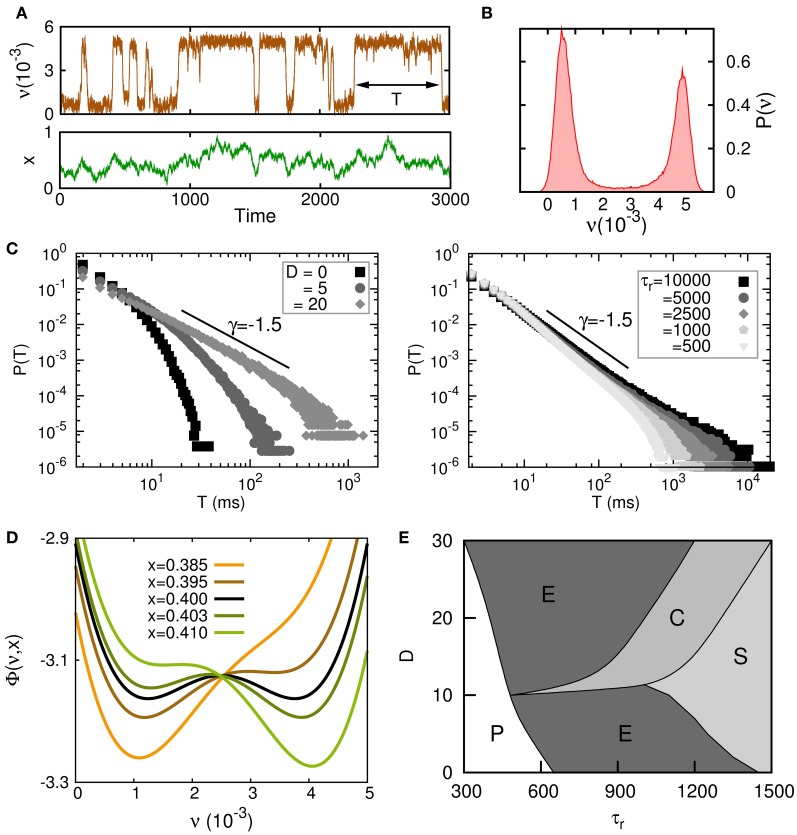
**Criticality in up–down transitions. (A)** Typical times series for the neuron population rate variable ν(*t*) and the mean depression variable *x*(*t*) in the neuron population when irregular up–down transitions emerge. Parameter values were *J* = 1.2 V, τ_*r*_ = 1000 τ_ν_ *U* = 0.6, *D* = 0, δ = 0.3, and ν_*m*_ = 5 · 10^−3^. **(B)** Histogram of the same time series for ν(*t*) which presents bimodal features corresponding to two different levels of activity. **(C)** Transitions from exponential to power law behavior for the probability distribution for the permanence time in the up or down state *P*(*T*) when parameters *D* (left panel) and τ_*r*_ (right panel) are varied. Model parameters were the same than in panel **(A)** except that *J* = 1.1 V in the left panel and *U* = 0.04 and *D*/τ_*r*_ = 0.02/τ_ν_ in the right panel. **(D)** A variation of *x*(*t*) induces a change in the shape of the potential function Φ—driving the dynamics of the rate variable ν(*t*)—which causes transitions between the up and down states. Parameters were the same than in panel **(A)** except that *J* = 1.1 V. **(E)** Complete phase diagram (*D*, τ_*r*_), for the same set of parameters than in panel **(D)**, where different phases characterize different dynamics of ν(*t*), *x*(*t*) (see main text for the explanation).

A complete analysis of this model, both theoretically and by numerical simulations, shows the appearance of complex transitions between high (up) and low (down) neural activity states driven by the synaptic noise *x*(*t*), with permanence times in the up state distributed according to a power-law for some range of the synaptic dynamic parameters. The main results of this study are summarized in Figure 4. On Figure 4A, a typical time series of the temporal behavior of the mean neural activity ν(*t*) of the system in the regime in which irregular up–down transitions occur is depicted. In Figure 4B, the histogram of ν(*t*) for this time series shows a clear bimodal shape corresponding to the two only possible states for ν(*t*). Figure 4C shows how the parameters τ_*r*_ and *D*, that control the stochastic dynamics of *x*(*t*), also are relevant for the appearance of power law distributions *P*(*T*) for the permanence time in the up or down state *T*. As is outlined in (Mejias et al., 2010), the dynamics can be approximately described in an adiabatic approximation, in which the neuron dynamics is subject to an effective potential Φ. Figure 4D shows how Φ changes for different values of the mean synaptic depression *x*. For relatively small *x* (orange and brown lines) all synapses in the population have a strong degree of depression and the population has a small level of activity, that is, the global minimum of the potential function is the low-activity state (the down state). On the other hand, when synapses are poorly depressed and *x* takes relatively large values (dark and light green lines) the neuron activity level is high and the potential function has its global minimum in a high-activity state (up state). For intermediate values of *x* (black line) the potential becomes bistable. Figure 4E shows the complete phase diagram of the system and illustrates the regions in the parameter space (*D*, τ_*r*_) where different behaviors emerge. In the phase (P) no transition between a high-activity state and low-activity state occurs. In phase (E) transitions between up and down states are exponentially distributed. The phase (C) is characterized by the emergence of power-law distributions *P*(*T*), and therefore is the most intriguing phase since it could be associated to a critical state. Finally, phase (S) is characterized by a highly fluctuating behavior of both ν(*t*) and *x*(*t*). In fact, ν(*t*) is behaving as a slave variable of *x*(*t*) and, therefore, it presents the dynamical features of the dynamics (Equation 16), which has some similarities with those of colored noise for *U* small. In fact for *U* = 0, and making the change *z*(*t*) = *x*(*t*) − 1 the dynamics (Equation 16) transforms in that for an Ornstein–Uhlenbeck (OU) process (van Kampen, 1990).

From these studies, we can conclude that the experimentally observed large fluctuations in up and down permanence times in the cortex can be explained as the result of sufficiently noisy dynamical synapses (large *D*) with sufficiently large recovery times (large τ_*r*_). Static synapses (τ_*r*_ = 0) or dynamical synapses in the absence of noise (*D* = 0) cannot account for this behavior, and only exponential distributions for *P*(*T*) emerge in this case.

## 5. Stochastic multiresonance

In section 2 we mentioned that short-term synaptic plasticity induces the appearance of dynamic memories as the consequence of the destabilization of memory attractors due to synapse fatigue. The synaptic fatigue in turn is due to strong neurotransmitter vesicle depletion as the consequence of high frequency presynaptic activity and large neurotransmitter recovering times. Also, we concluded that this fact induces a high sensitivity of the system to respond to external stimuli, even if the stimulus is very weak and in the presence of noise. The source of the noise can be due to the neural dynamics as well as the synaptic transmission. It is the combination of non-linear dynamics and noise that causes the enhanced sensitivity to external stimuli. This general phenomenon is the so called *stochastic resonance* (SR) (Benzi et al., 1981; Longtin et al., 1991).

In a set of recent papers we have studied the emergence of SR in feed-forward neural networks with dynamic synapses (Mejias and Torres, 2011; Torres et al., 2011). We considered a post-synaptic neuron which receives signals from a population of *N* presynaptic neurons through dynamic synapses modeled by Equations (1, 2). Each one of these presynaptic neurons fires a train of Poisson distributed APs with a given frequency *f*_*n*_. In addition the postsynaptic neuron receives a weak signal *S*(*t*) which we can assume sinusoidal. In addition, we assume a stationary regime, where the dynamic synapses have reached their asymptotic values $u_{\infty }=\frac{U+U\tau_{\text{fac}}f_{n}}{1+U\tau_{\text{fac}}f_{n}}$ and $x_{\infty }=\frac{1}{1+u_{\infty }\tau_{\text{rec}}f_{n}}$. If all presynaptic neurons fire independently the total synaptic current is a noisy quantity with mean Ī_*N*_ and variance σ^2^_*N*_ given by

(17) $$\begin{array}{l} {\bar{I}}_{N}=Nf_{n}\tau_{\text{in}}I_{p}\\ \sigma_{N}^{2}=\frac{1}{2}Nf_{n}\tau_{\text{in}}{\left(I_{p}\right)}^{2} \end{array}$$

with *I*_*p*_ = *A u*_∞_*x*_∞_ and *A* the synaptic strength. To explore the possibility of SR, we vary the firing frequency of the presynaptic population *f*_*n*_. The reason for this choice is that varying *f*_*n*_ changes the output variance σ^2^_*N*_ and *f*_*n*_ can also be relatively easily controlled in an experiment.

**Figure 5:**
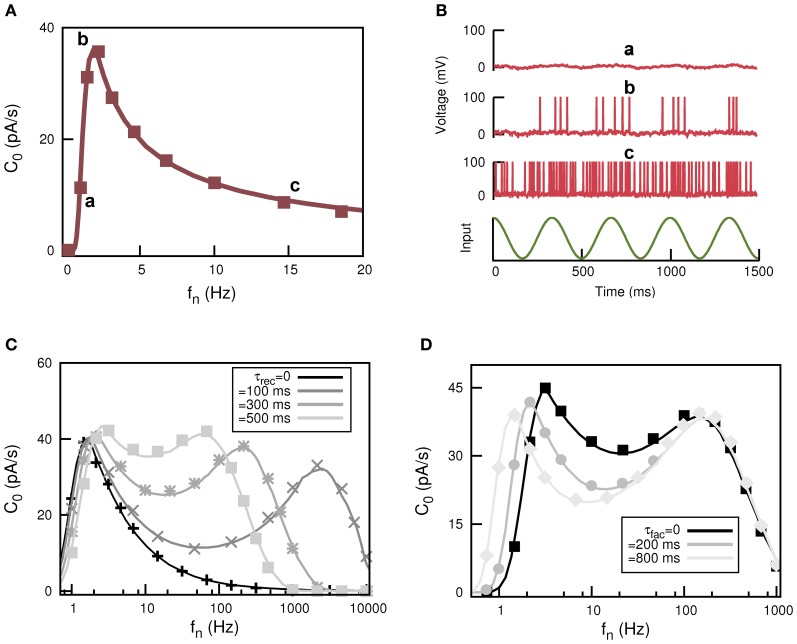
**Appearance of stochastic multiresonances in feed forward neural networks of spiking neurons with dynamic synapses. (A)** Behaviour of *C*_0_—defined in Equation (18)—as a function of *f*_*n*_ for static synapses showing the phenomenon of stochastic resonance. **(B)** Temporal behavior for the response of the postsynaptic neuron at each labeled position of the resonance curve in panel **(A)**. **(C)** Resonance curve for *C*_0_ when dynamic synapses are included. The most remarkable feature is the appearance of a two-peak resonance in the frequency domain, with the position of high frequency peak controlled by the particular value of τ_rec_. **(D)** The panel shows another interesting feature of the two-peak resonance curve for *C*_0_, that is, the control of the position of the low frequency peak by τ_fac_.

To quantify the amount of signal that is present in the output rate we use the standard input–output cross-correlation or *power norm* (Collins et al., 1995) during a time interval Δ*t* and defined as:

(18) $$C_{0}=\langle S(t)\nu (t)\rangle =\frac{1}{\Delta t}\int_{t}^{t+\Delta t}S(t)\nu (t)dt,$$

where ν(*t*) is the firing rate of the post-synaptic neuron. The behavior of *C*_0_ as a function of *f*_*n*_ for static synapses is depicted in Figure 5A which clearly shows a resonance peak at certain non-zero input frequency *f*_n_. The output of the postsynaptic neuron at the positions in the frequency domain labeled with “a,” “b,” and “c” is illustrated in Figure 5B and compared with the weak input signal. This shows how stochastic resonance emerges in this system. For low firing frequency (case labeled with “a”) in the presynaptic population the generated current is so small that the postsynaptic neuron only has sub-threshold behavior weakly correlated with *S*(*t*). For very large *f*_*n*_ (case labeled with “c”) both Ī_*N*_ and σ^2^_*N*_ are large and the postsynaptic neuron is firing all the time, so it can not detect the temporal features of *S*(*t*). However, there is an optimal value of *f*_*n*_ at which the postsynaptic neuron fires strongly correlated with *S*(*t*); in fact it fires several APs each time a maximum in *S*(*t*) occurs (case labeled with “b”).

This behavior dramatically changes when dynamic synapses are considered, as is depicted in Figures 5C,D. In fact, for dynamic synapses there are two frequencies at which resonance occurs. That is, short-term synaptic plasticity induces the appearance of stochastic multi-resonances (SMR). Interestingly, the position of the peaks is controlled by the parameters that control the synapse dynamics. For instance, in Figure 5C it is shown how for a fixed value of facilitation and increasing depression (increasing τ_rec_) the second resonance peak moves toward low values of *f*_*n*_ while the position of the first resonance peak remains unchanged. On the other hand, for a given value of depression, the increase of facilitation time constant τ_fac_ moves the first resonance peak while the position of the second resonance peak is unaltered (see Figure 5D). This clearly demonstrates that in actual neural systems synapses with different levels of depression and facilitation can control the signal processing at different frequencies.

The appearance of SMR in neural media with dynamic synapses is quite robust: SMR also appears when the post-synaptic neuron is model with different types of spiking mechanisms, such as the FitzHugh–Nagumo (FHN) model or the integrate and fire model (IF) with an adaptive threshold dynamics (Mejias and Torres, 2011). SMR also appears with more realistic stochastic dynamic synapses and more realistic weak signals such as a train of inputs with small amplitude and short durations distributed in time according to a rate modulated Poisson process (Mejias and Torres, 2011).

The physical mechanism behind the appearance of SMR is the existence of a non-monotonic dependence of the synaptic current fluctuations with *f*_*n*_—due to the dynamic synapses—together with the existence of an adaptive threshold mechanism in the postsynaptic neuron to the incoming synaptic current. In this way, the distance in voltage between the mean post-synaptic sub-threshold voltage and the threshold for firing remains constant or decreases very slowly for increasing presynaptic frequencies. This implies the existence of two values of *f*_*n*_ at which current fluctuations are enough to induce firing in the post-synaptic neuron [see Mejias and Torres (2011) for more details].

**Figure 6:**
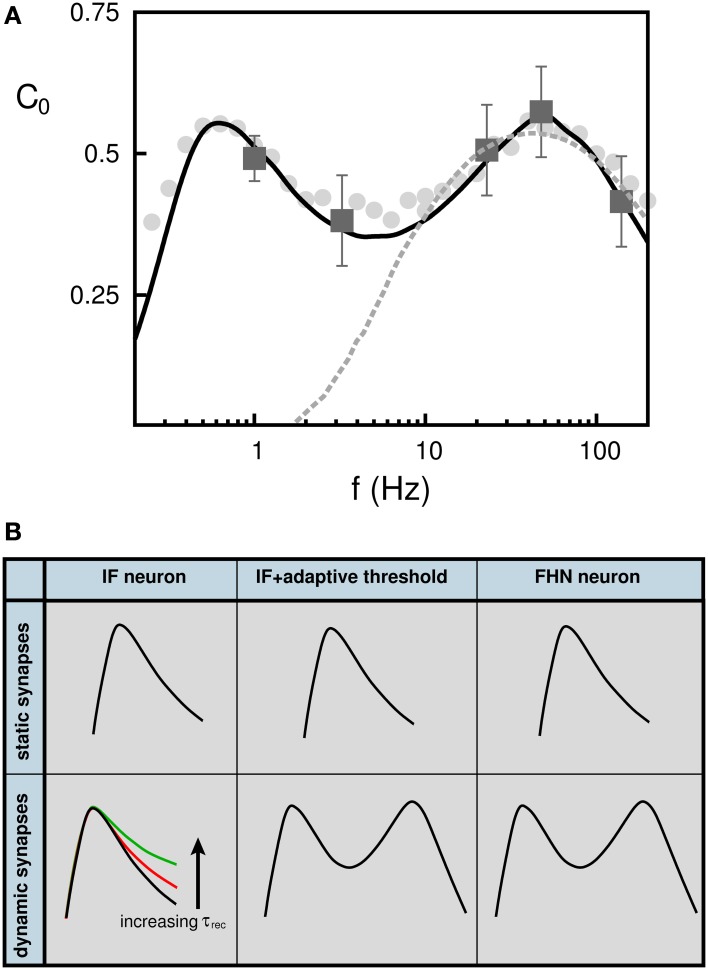
**(A)** Appearance of stochastic multi-resonance in experiments in the brain. Dark gray square symbols represent the values of *C*_0_ obtained in the experiments performed in the human auditory cortex. Dashed light gray line corresponds to best model prediction using a neuron with fixed threshold (Yasuda et al., 2008). Solid black line correspond to our model consisting of a FHN neuron and depressing synapses. Gray filled circle symbols shows *C*_0_ when the weak signal is a train of (uncorrelated) Poisson pulses instead of the sinusoidal input (solid line). **(B)** Schematic overview showing the neuron and synapse mechanisms needed for the appearance of stochastic multi-resonances in feed-forward neural networks. (see (Torres et al., 2011) for more details).

In light of these findings, we have reinterpreted recent SR experimental data from psycho-physical experiments on human blink reflex (Yasuda et al., 2008). In these experiments the neurons responsible for the blink reflex receive inputs from neurons in the auditory cortex, which are assumed to be uncorrelated due to the action of some external source of white noise. The subject received in addition a weak signal in the form of a periodic small air puff into the eyes. The authors measured the correlation between the air puff signal and the blink reflex and their results are plotted in Figure 6A (dark gray square error-bar symbols). They used a feed-forward neural network with a postsynaptic neuron with IF dynamics with fixed threshold to interpret their findings (light-gray dashed line). With this model, only the high-frequency correlation points can be fitted. Using instead a FHN model or an IF with adaptive threshold dynamics, we were able to fit all experimental data points (black solid line). The SMR is also observed with more realistic rate-modulated weak Poisson pulses (light-gray filled circles) instead of the sinusoidal input (black solid line). Both model predictions are consistent with the SMR that is observed in this experiments. In Figure 6B we summarize the conditions that neurons and synapses must satisfy for the emergence of SMR in a feed forward neural network.

## 6. Relation with other works

The occurrence of non-fixed point behavior in recurrent neural networks due to dynamic synapses has also been reported by others (Senn et al., 1996; Tsodyks et al., 1998; Dror and Tsodyks, 2000). These studies differ from our work because one assumes continuous deterministic neuron dynamics (instead of binary and stochastic, as in our work). The oscillations observed in these networks do not have the rapid switching behavior as we observe and seem unrelated to the metastability that we have found in our work.

In addition, it has been reported that oscillations in the firing rate can be chaotic (Senn et al., 1996; Dror and Tsodyks, 2000) and present some intermittent behavior that resembles observed patterns of EEG. The chaotic regime in these continuous models seems unrelated to the existence of fixed point behavior and most likely understood as a generic feature of non-linear dynamical systems.

It is worth noting that for each neuron, the effect of dynamic synapses is modeled through a single variable *x*_*i*_ that multiplies the synaptic strength *w*_*ij*_ for all synapses that connect to *i*. There is one depression variable per *neuron* and not per connection. As a result, one can obtain the same behavior of the network by interpreting *x*_*i*_ as implementing a dynamic firing thresholds (Horn and Usher, 1989) instead of a dynamic synapse.

The switching behavior that we described in this paper, is somewhat similar to the neural network with chaotic neurons that displays a self-organized chaotic transition between memories (Tsuda et al., 1987; Tsuda, 1992).

The possible interpretation of the switching behavior as up/down cortical transitions is controversial, because similar cortical oscillations can be generated without synaptic dynamics, where the up state is terminated because of hyperpolarizing potassium ionic currents (Compte et al., 2003). However, a very recent study has focused on the interplay between synaptic depression and these inhibitory currents and concludes that synaptic depression is relevant for maintaining the up state (Benita et al., 2012). The reason for that counterintuitive behavior is that synaptic depression decreases the firing rate in the up state which also decreases the effect of the hyper-polarizing potassium currents and, as a consequence, the prolongation of the up state.

Related also is a recent study on the effect of dynamic synapses on the emergence of a coherent periodic rhythm within the Up state which results in the phenomenon of *stochastic amplification* (Hidalgo et al., 2012). It has been shown that this rhythm is an emergent or collective phenomenon given that individual neurons in the up state are unlocked to such a rhythm.

The relation between dynamic synapses and storage capacity has also been studied by others. For very sparse stored patterns (*a* « 1) it has been shown that storage capacity decreases with synaptic depression (Bibitchkov et al., 2002), in agreement with our findings. On the other hand, it has been reported that the basin of attraction of the memories are enlarged by synaptic depression (Matsumoto et al., 2007) and these are even enlarged more when synaptic facilitation is taken into account (Mejias and Torres, 2009).

(Otsubo et al., 2011) reported a theoretical and numerical study on the role of short-term depression on memory storage capacity in the presence of noise, showing that noise reduces the storage capacity (as is also the case for static synapses). (Mejias et al., 2012) shows the important role of facilitation to enlarge the regions for memory retrieval even in the presence of high noise.

In the last decade there has been some discussion whether neural systems, or even the brain as a whole, can work in a critical state using the notion of self-organized criticality (Beggs and Plenz, 2003; Tagliazucchi et al., 2012). As we stated in section 4, the combination of colored synaptic noise and short-term depression can cause power-low distributed permanence times in the Up and Down states, which is a signature of criticality. The emergence of critical phenomena as a consequence of dynamic synapses has also been explored by others (Levina et al., 2007, 2009; Bonachela et al., 2010; Millman et al., 2010).

Finally, it is worth mentioning a recent work that has investigated the formation of spatio-temporal structures in an excitatory neural network with depressing synapses (Kilpatrick and Bressloff, 2010). As a result of dynamic synapses, robust complex spatio-temporal structures, including different types of travelling waves, appear in such a system.

## 7. Conclusions

It is well-known that during transmission of information, synapses show a high variability with a diverse origin, such as the stochastic release and transmission of neurotransmitter vesicles, variations in the Glutamate concentration through synapses and the spatial heterogeneity of the synaptic response in the dendrite tree (Franks et al., 2003). The cooperative effect of all these mechanisms is a noisy post-synaptic response which depends on past pre-synaptic activity. The strength of the postsynaptic response can decrease or increase and can be modeled as dynamical synapses.

In a large number of papers, we have studied the effect of dynamical synapses in recurrent an feed-forward networks, the result of which we have summarized in this paper. The main findings are the following:

**Dynamic memories:** Classical neural networks of the Hopfield type, with symmetric connectivity, display attractor dynamics. This means that these networks act as memories. A specific set of memories can be stored as attractors by Hebbian learning. The attractors are asymptotically stable states. The effect of synaptic depression in these networks is to make the attractors lose stability. Oscillatory modes appear where the network rapidly switches between memories. Instead, the permanence time to stay in a memory can have any positive value and becomes infinite in the regime where memories are stable. Thus, the recurrent network with dynamical synapses implements a form of dynamical memory.
**Input sensitivity:** The classical Hopfield network is relatively insensitive to external stimuli, once it has converged into one of its stable memories. Synaptic depression improves the sensitivity to external stimuli, because it destabilizes the memories. In addition, synaptic facilitation further improves the sensitivity of the attractor network to external stimuli.
**Storage capacity:** The storage capacity of the attractor neural network, i.e., the maximum number of memories that can be stored in a network, is proportional to the number of neurons *N* and scales as *P*_max_ = α*N* with α = 0.138. Synaptic depression causes a decrease of the maximum storage capacity but facilitation allows to recover the capacity of the network with static synapses under some conditions.
**Up and down states:** The emergence of dynamic memories has been related to the well-known up–down transitions observed in local-field recording in the cortex. We demonstrated that the observed distributions of permanence times can be explained by a stochastic synaptic dynamics. Scale free permanence time distributions could signal a critical state in the brain.
**Stochastic multiresonance:** Whereas static synapses in a stochastic network give rise to a single stochastic resonance peak, dynamical synapses produce a double resonance. This phenomenon is robust for different types of neurons and input signals. Thus, dynamic synapses may explain recently observed SMR in psychophysical experiments. SMR also seems to occur in recurrent neural networks with dynamic synapses as it has been recently reported (Pinamonti et al., 2012). This work demonstrates the relevant role of short-term synaptic plasticity for the appearance of the SMR phenomenon in recurrent networks, although the exact underlying mechanism behind it is slightly different than in the case described here, namely feed-forward neural networks.

It is important to point out that although the phenomenology reported in this review has been obtained using different models, *all* the reported phenomena can be also derived in a single model consisting in a network of binary neurons with dynamic synapses as described in section 1. The phenomena reported in sections 2 and 3 have in fact been obtained using this model and the phenomenon of stochastic multiresonance (section 5) has been reported recently in such a model by Pinamonti et al. (2012). The results on critical up and down states that are reported in section 4 have been obtained in a mean-field model that can be derived from the same binary model and by assuming in addition sparse neural activity and sparse connectivity, which increases the stochasticity in the synaptic transmission through the whole network.

In addition, our studies show that the reported phenomena are robust to detailed changes in the model, such as replacing the binary neurons by graded response neurone or integrate-and-fire neurone.

### Conflict of interest statement

The authors declare that the research was conducted in the absence of any commercial or financial relationships that could be construed as a potential conflict of interest.
